# Warfarin-induced skin necrosis concurrent with dengue fever: a case report highlighting diagnostic and management challenges in a conflict-affected region

**DOI:** 10.1093/omcr/omaf047

**Published:** 2025-05-28

**Authors:** Alsadig Suliman, Siddig Ali, Hiba Suliman, Shorouq Mohammed Ali, Reem Mohamed Osman

**Affiliations:** Department of General Surgery, Sudan Medical Specialization Board, Isbitalia Street, Downtown, Khartoum, Khartoum 13315, Sudan; Department of General Surgery, Sudan Medical Specialization Board, Isbitalia Street, Downtown, Khartoum, Khartoum 13315, Sudan; Department of General Surgery, Wad Medani College of Medical Sciences & Technology, Darja Street, Wad Madani, Wad Madani 21111, Sudan; Department of General Surgery, Wad Medani College of Medical Sciences & Technology, Darja Street, Wad Madani, Wad Madani 21111, Sudan; Department of General Surgery, Al-Neelain University, Zubeir Pasha Street, Downtown, Khartoum, Khartoum 11115, Sudan

**Keywords:** warfarin-induced skin necrosis (WSN), mechanical heart valve, anticoagulation, resource-limited settings, conflict-affected areas, surgical debridement

## Abstract

Warfarin-induced skin necrosis (WSN) is a rare but serious complication of anticoagulation therapy. This case report describes a 34-year-old male with mechanical heart valves on long-term warfarin therapy who developed WSN after self-medicating with a doubled warfarin dose following a two-week interruption due to limited healthcare access in a conflict-affected region. Concurrently, he was diagnosed with dengue fever, further complicating anticoagulation management due to thrombocytopenia. Prompt discontinuation of warfarin, intravenous vitamin K administration, and delayed initiation of enoxaparin after platelet recovery were key aspects of treatment. Surgical debridement of necrotic skin lesions was performed, resulting in stabilization and recovery. This case highlights the unique diagnostic and management challenges of WSN in the setting of a concurrent dengue infection, especially in resource-limited settings and conflict-affected areas. Early recognition and tailored intervention are essential to prevent severe complications and improve outcomes.

## Introduction

Warfarin, a vitamin K antagonist, is widely used for preventing thromboembolic events in patients with mechanical heart valves and atrial fibrillation. By inhibiting the synthesis of vitamin K-dependent clotting factors (II, VII, IX, and X), as well as proteins C and S, warfarin requires frequent INR monitoring due to its narrow therapeutic index [1]. A transient hypercoagulable state may occur early in therapy due to the rapid depletion of proteins C and S, increasing the risk of complications such as warfarin-induced skin necrosis (WSN) [2].

In resource-limited and conflict-affected regions, managing anticoagulation therapy is further complicated by treatment interruptions, poor medication adherence, and limited access to healthcare. Additionally, in tropical regions, the coagulopathic effects of endemic diseases like dengue fever add another layer of complexity to anticoagulation management [3]. These combined factors create unique therapeutic dilemmas, particularly when balancing thrombosis prevention with bleeding risks.

This case report highlights the challenges of managing WSN in a patient with concurrent dengue fever in a conflict-affected setting with limited diagnostic and treatment options [4]. It underscores the urgent need for adapted anticoagulation strategies and evidence-based guidelines to optimize patient outcomes.

## Case presentation

**Figure 1:**
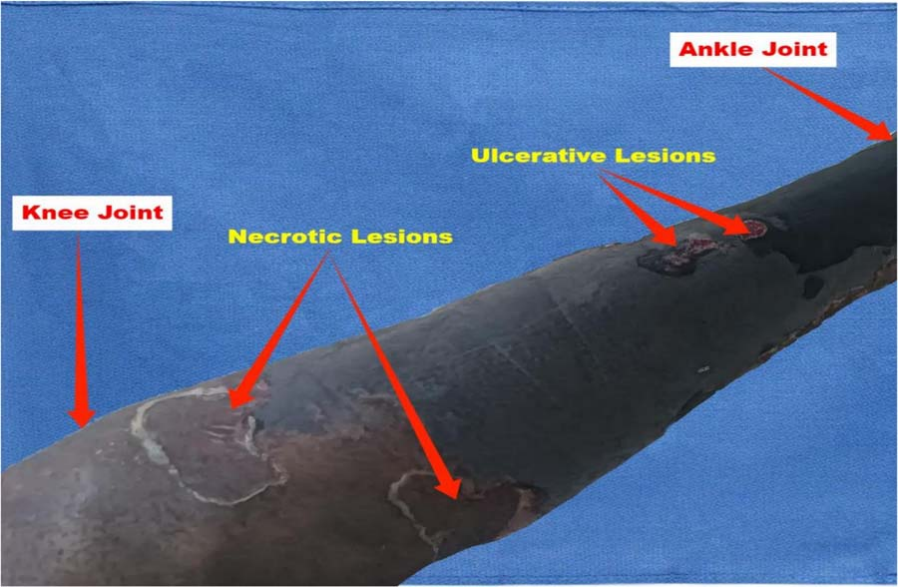
Anterolateral view of the right leg. Shows necrosis with central eschar, surrounding erythema, and ulcerative lesions. Clear demarcation.

**Figure 2:**
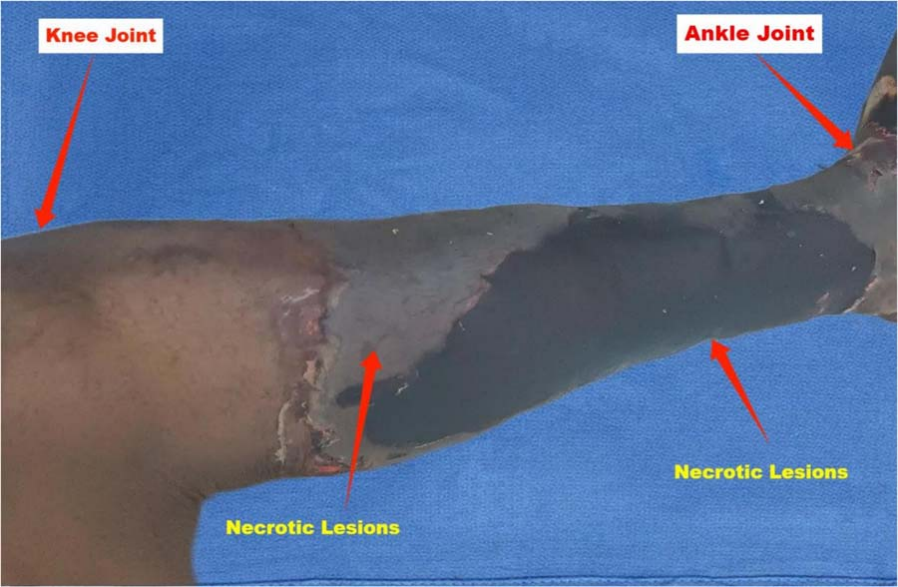
Medial view of the left leg. Displays necrotic lesions with dark eschar and inflamed, sharp boundary between viable and necrotic area.

A 34-year-old male presented to the emergency department with painful black discoloration on both lower limbs (Figs 1 and 2). His medical history was significant for mechanical heart valves, and he was on long-term warfarin therapy (5 mg daily), maintaining a target INR of 2. The patient reported discontinuing anticoagulation for two weeks due to limited access to healthcare in his conflict-affected region. Concerned about worsening joint pain and fever, he self-medicated by doubling his warfarin dose for three days without consulting a healthcare provider. Upon presentation, he complained of generalized body aches, headache, and fever.

**Figure 3:**
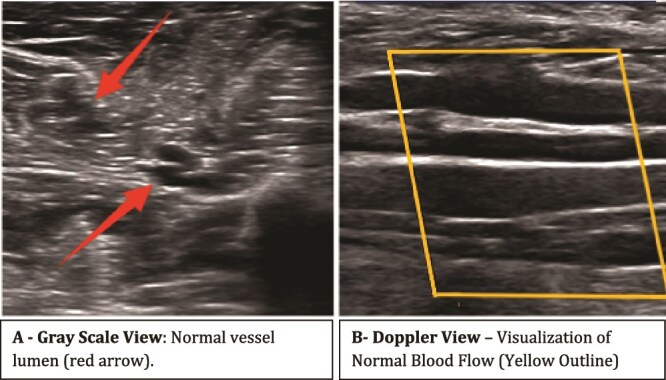
Doppler ultrasound of right leg vessels.

**Figure 4:**
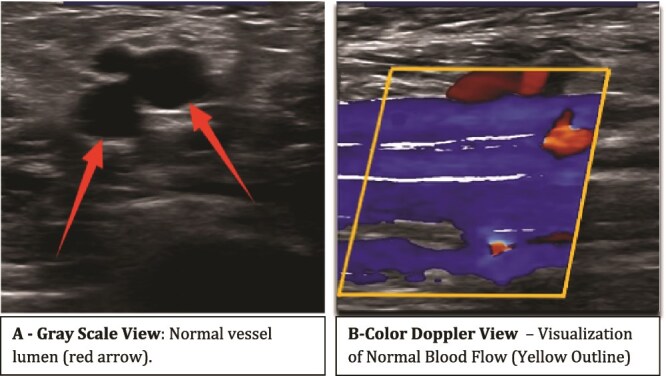
Doppler ultrasound of left leg vessels.

Investigations (Table 1) revealed significant thrombocytopenia, with a platelet count of 48 000/μl. The patient was febrile and came from a dengue-endemic region, prompting dengue serology (IgM), which tested positive. Additionally, there was suspicion of an underlying protein C or S deficiency, though confirmatory testing was unavailable. A Doppler ultrasound ruled out deep vein thrombosis (DVT) and confirmed good arterial supply (Figs 3 and 4).

**Table 1:** Serial investigations of the patient.

Normal Range	WBC (×10^3^/μl)	Hb (g/dl)	PLT (×10^3^/μl)	Dengue IgM	INR	PT (sec)	APTT (sec)	Creatinine (mg/dl)
	4.5–11	13–17	150–450	- ve	0.9–1.2	11–15	25–35	0.6–1.2
Day 1	2.4↓	12.5	48↓	+	5.5↑	16↑	38↑	1.2
Day 3	3.0↓	11.5	68↓	+	4.0↑	20↑	35↑	1.1
Day 7	3.5	11.5	85↓	−	3.0	18	30	1.0
Day 10	4.8	12.5	160	−	2.0	14	28	0.9
Day 14	5.1	12.9	273	−	1.5	12	26	0.8
Day 28	6.0	14.3	250	−	1.0	11	25	0.7

Due to severe thrombocytopenia and the associated high risk of bleeding, a skin biopsy was not performed. Instead, the clinical diagnosis of WSN was made based on the patient’s history of warfarin overdose, the rapid progression of necrotic skin lesions, and the exclusion of alternative diagnoses such as DVT and disseminated intravascular coagulation (DIC). The patient’s favorable response to treatment further supported this diagnosis. Both blood and wound cultures returned negative. Once the platelet count improved, subcutaneous enoxaparin (40 mg daily) was initiated.

Dengue fever was managed conservatively with fluid resuscitation and close monitoring of platelet counts. Necrotic skin was debrided on day 7 after stabilizing platelet counts. The patient’s INR was successfully stabilized between 2–3, and he was counseled on the importance of avoiding self-medication and ensuring regular follow-ups. Two weeks post-discharge, his skin lesions showed significant improvement. He was discharged with a follow-up plan to monitor his INR, platelet count, and skin recovery.

## Discussion

WSN is a rare but serious complication of anticoagulation therapy, occurring in approximately 0.01%–0.1% of patients [5]. It typically develops within 2–5 days of initiating warfarin, particularly in individuals with protein C or S deficiency, due to a transient hypercoagulable state [6]. WSN presents as painful purpuric lesions in subcutaneous fat-rich areas such as the breasts, thighs, and buttocks, progressing to necrosis and eschar formation [7]. Diagnosis is primarily clinical, supported by histopathology showing dermal vessel thrombosis without vasculitis [8]. Management includes immediate discontinuation of warfarin, administration of intravenous vitamin K, initiation of heparin bridging therapy, and surgical debridement in severe cases [9]. Dengue fever, a mosquito-borne viral illness, poses a significant challenge in anticoagulation management due to its profound effects on coagulation homeostasis. It is characterized by thrombocytopenia, endothelial dysfunction, and reduced levels of protein C, protein S, and antithrombin III, increasing the risk of both bleeding and thrombosis [10]. The coexistence of WSN and dengue fever exacerbates hemostatic instability, creating a complex therapeutic dilemma requiring meticulous risk–benefit assessment.

This case of WSN concurrent with dengue fever presents unique challenges compared to previously reported cases. Nsaful et al. described WSN following a warfarin overdose in a patient with renal failure and sepsis, requiring dialysis and multiple debridements [9]. In contrast, our patient developed WSN in the setting of dengue-associated thrombocytopenia, which significantly influenced anticoagulation decisions. Zahra et al. reported WSN in a 14-year-old female despite enoxaparin bridging and a normal clotting profile [7], reinforcing that WSN can occur even in the absence of confirmed thrombophilia, as seen in our case.

Srinivasan et al. documented WSN with venous limb gangrene in the setting of heparin-induced thrombocytopenia (HIT), where warfarin use exacerbated a hypercoagulable state [11]. Similarly, our patient’s coagulopathy, influenced by dengue fever-induced thrombocytopenia, may have contributed to an imbalance in hemostasis, complicating anticoagulation management and increasing the risk of WSN. Kakagia et al. reported WSN in a patient with no documented thrombophilia and a normal clotting profile [12], highlighting a diagnostic challenge that parallels our case. Sklar et al. described WSN in a patient with prior warfarin exposure, who developed necrosis after recommencing warfarin [13]. This aligns with our patient’s history of long-term warfarin use, with WSN developing after self-medicating with a doubled dose.

This case is, to our knowledge, the first documented instance of WSN occurring concurrently with dengue fever, presenting a unique anticoagulation challenge. Unlike prior cases, the interplay of warfarin toxicity and dengue-induced thrombocytopenia created a complex hemostatic imbalance, requiring meticulous anticoagulation adjustments and delayed surgical debridement. The patient’s treatment disruption due to conflict-related healthcare inaccessibility underscores the risks of self-medication in resource-limited settings [5–9, 13]. This case highlights the need for tailored anticoagulation strategies, patient education, and evidence-based guidelines for managing anticoagulation in tropical infections and conflict-affected regions.
